# Three-Dimensional Assessment of Maxillary Stability Using Customized Plates in Orthognathic Surgery: A Retrospective Cohort Study

**DOI:** 10.3390/cmtr19020027

**Published:** 2026-06-09

**Authors:** Leonardo Aguilar, Juan Pablo Vargas Buratovic, Valentina Matamala Ibaceta, Felipe Merchan, Alberto Fuhrer, Ximena Toledo

**Affiliations:** 1 Department of Oral and Maxillofacial Surgery, RedSalud Providencia Clinic, Santiago 7500995, Chile; drleonardoaguilar@gmail.com (L.A.); maxilofacial.albertofuhrer@gmail.com (A.F.); 2 Oral and Maxillofacial Surgeon, Dr. Sotero del Rio Hospital, Santiago 8160000, Chile; felipe43083@yahoo.com; 3 Resident of Oral and Maxillofacial Surgery, Pontifical Catholic University, Santiago 7820436, Chile; 4 Department of Dentistry, University of the Andes, Santiago 7550000, Chile; vmatamalaibaceta@gmail.com; 5 Department of Orthodontics, Faculty of Dentistry, University of Chile, Santiago 8380492, Chile; xtoledo@clinicamanquehue.cl

**Keywords:** orthognathic surgery, patient-specific implant, virtual surgical planning, skeletal stability, Le Fort I osteotomy

## Abstract

Patient-specific implants (PSIs) in orthognathic surgery offer optimal intraoperative accuracy. However, evidence regarding their postoperative skeletal stability, specifically comparing distinct fixation designs and segmentation patterns, remains limited. We present a retrospective cohort study that evaluated 64 adult patients undergoing customized maxillary orthognathic surgery between January 2020 and June 2025. The primary predictor variables were fixation design (conventional customized plates vs. minimally invasive plates) and maxillary segmentation (monoblock vs. multisegmental). The outcome variable was 3D skeletal stability, measured as linear displacement between preoperative planning and 6-month postoperative imaging. Non-parametric tests compared displacements and clinical instability rates (defined as ≥2.0 mm). Mann–Whitney tests compared landmark displacements, Fisher’s exact tests compared proportions with ≥2.0 mm displacement, and ORs with 95% CIs were computed (α = 0.05). Analysis of 64 patients revealed that median displacement across landmarks ranged from 0.7 to 4.28 mm and 28.1% exhibited displacement ≥ 2.0 mm, primarily in molar and canine regions. While overall instability rates did not differ significantly between single-segment and multisegmental osteotomies (*p* = 0.28), multisegmental cases showed significantly higher displacement at the left canine (*p* = 0.027). Plate design was not associated with skeletal instability (*p* = 0.88), suggesting that minimally invasive plates provide comparable stability to conventional designs. Customized maxillary plates provide reliable postoperative stability with median displacements within clinically acceptable limits (<2 mm). Minimally invasive PSI designs offer stability comparable to conventional extended designs. However, localized instability in multisegmental cases suggests a need for careful biomechanical management regardless of the fixation method used.

## 1. Introduction

Orthognathic surgery aims to restore facial balance and functional occlusion through the three-dimensional repositioning of the maxilla and mandible. The long-term success of this procedure depends on both the precision of skeletal movements and the postoperative stability of bone segments. Conventional workflows based on two-dimensional cephalometry, dental casts, and intermediate splints have demonstrated inherent inaccuracies derived from articulator mounting, manual model surgery, and intraoperative transfer errors [1,2]. These limitations may affect not only the accuracy of jaw positioning but also the predictability of long-term stability.

Over the past two decades, virtual surgical planning (VSP) and computer-aided design and manufacturing (CAD/CAM) technologies have revolutionized orthognathic surgery, allowing precise digital simulation of osteotomies, virtual repositioning of bone segments, and fabrication of patient-specific devices for intraoperative guidance [3,4]. This digital workflow enables improved surgical accuracy, reduced planning time, and enhanced communication between surgeons and orthodontists [5,6,7]. Consequently, the development of patient-specific osteosynthesis systems—comprising customized cutting guides and fixation plates—has gained increasing clinical acceptance [8,9].

Multiple studies have demonstrated that patient-specific plates provide high accuracy in transferring virtual plans to the operative field. Rückschloß et al. (2021) reported mean translational deviations below 1 mm in Le Fort I osteotomies using 3D-printed cutting guides and plates [10], while Lin et al. (2024) observed mean vertical and sagittal errors of approximately 1 mm and rotational deviations below 2°, confirming clinically acceptable precision [11]. Similarly, Hanafy et al. (2020) found that CAD/CAM-guided Le Fort I osteotomies achieved comparable or superior accuracy to conventional splint-based techniques, with reduced intraoperative time and fewer adjustments required [12].

Beyond accuracy, customized fixation systems offer workflow and biomechanical advantages. Plates are pre-shaped to match the patient’s anatomy, with pre-defined drill trajectories that minimize intraoperative bending, preserve root and neurovascular integrity, and ensure reproducible fixation [13]. Furthermore, the waferless approach eliminates the need for intermaxillary splints, reducing occlusal interferences and improving visualization during maxillary repositioning [14]. In a clinical series of 43 patients, Fleury et al. (2022) reported a significant reduction in operative time when using customized maxillary plates compared to conventional fixation, with no increase in complication rates [9].

However, despite promising outcomes, the literature remains limited regarding postoperative skeletal stability following fixation with patient-specific plates. Most studies focus on intraoperative accuracy, short-term outcomes, or technical feasibility rather than long-term positional maintenance [15,16,17]. Moreover, available reports are often single-center series with heterogeneous protocols, small samples, or variable follow-up intervals. Quantifying postoperative stability after CAD/CAM-based fixation is therefore essential to validate its biomechanical reliability and clinical superiority over conventional methods.

The present study aims to evaluate postoperative maxillary stability in patients who underwent orthognathic surgery with customized fixation plates. Using standardized three-dimensional image superimposition at six months postoperatively, we compared linear displacements across multiple maxillary landmarks according to the number of maxillary segments and plate design. This study seeks to provide high-level evidence regarding the accuracy and stability of customized osteosynthesis systems in orthognathic surgery.

## 2. Materials and Methods

A retrospective observational study was conducted, including all adult patients who underwent customized orthognathic surgery between January 2020 and June 2025 at a private practice single tertiary center. Inclusion criteria comprised healthy adults classified as ASA I, aged between 20 and 40 years, presenting with Class II or Class III dentofacial deformity, with or without associated facial asymmetry. All patients were candidates for bimaxillary orthognathic surgery based on functional and esthetic criteria. Exclusion criteria included craniofacial syndromes, cleft lip and palate, previous facial trauma, systemic disease, or incomplete radiological data.

### 2.1. Virtual Surgical Planning and Fixation-System Classification

All cases underwent 3D virtual surgical planning using preoperative CT imaging and intraoral digital scans integrated in CAD/CAM software (PROPLAN CMF, version 3.0.1, Materialise, Leuven, Belgium and 3-Matic software version 18.0). The virtual plan defined osteotomy design, 3D repositioning, and patient-specific maxillary plate design.

Customized titanium fixation systems were fabricated for the maxilla using patient-specific design and selective laser melting, utilizing commercial systems from either Orthomax (Santiago, Chile) or Prototipos Médicos e Industriales SPA (PROTAICO; Santiago, Chile). No intermediate occlusal splint was used for maxillary repositioning (waferless protocol) [10,11].

**Figure 1:**
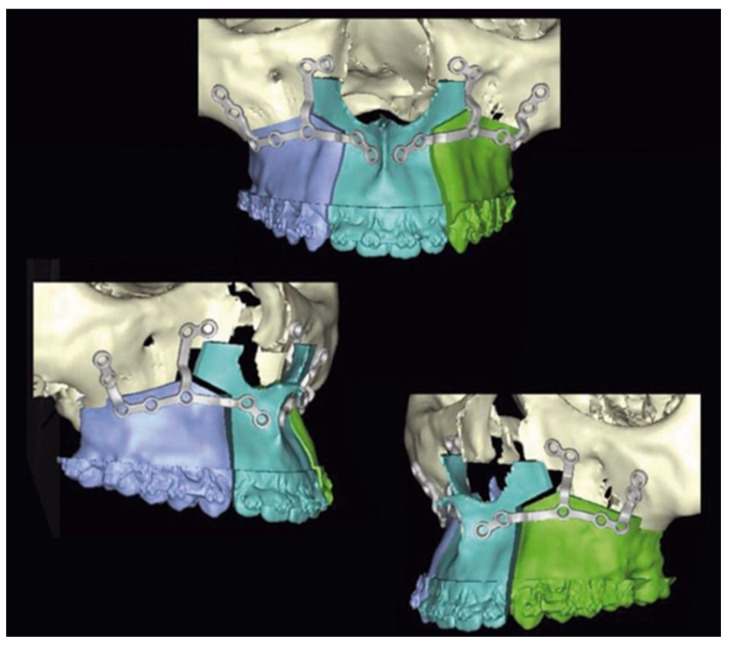
Virtual planning of conventional customized plates.

**Figure 2:**
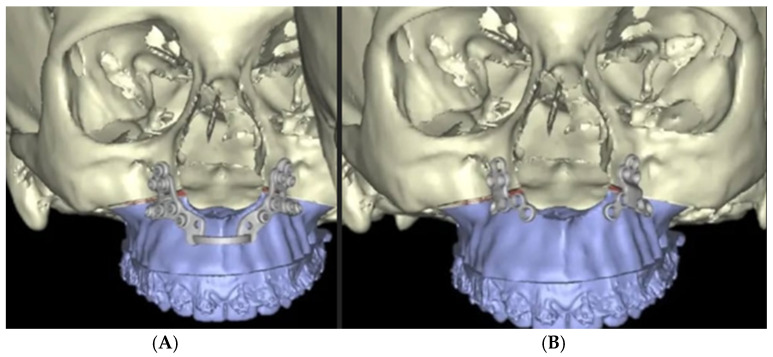
3D planning of minimally invasive customized plates: (**A**) Cutting guides; (**B**) Plates.

Customized plates were classified according to fixation pattern:

1. Conventional customized plates: These extend over both the nasomaxillary and zygomaticomaxillary buttresses (Figure 1).

2. Minimally invasive customized plates: Limited to the nasomaxillary buttress, these are designed to reduce surgical exposure and hardware volume (3), as shown in Figure 2.

### 2.2. Surgical Procedure

**Figure 3:**
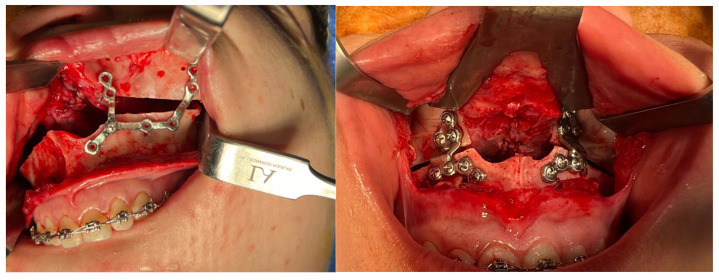
Customized anterior and posterior support plate. Customized anterior support plate.

All operations were performed by the same surgical team under general anesthesia with nasotracheal intubation. A maxilla-first orthognathic approach was used [4,5]. Following a vestibular incision, a Le Fort I osteotomy was executed with piezosurgery or a reciprocating saw according to bone thickness. The maxilla was down-fractured, mobilized, and positioned according to the virtual plan using patient-specific cutting guides, then fixated with the assigned customized plates (Figure 3) and screws.

**Figure 4:**
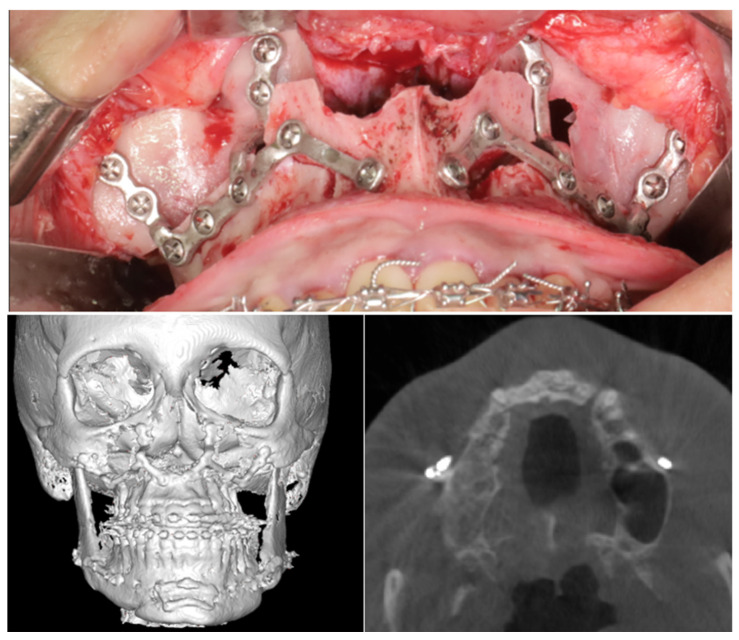
Intraoperative view of a three-piece Le Fort I osteotomy stabilized with an anterior and posterior support plate, and corresponding postoperative 3D reconstruction and axial images.

**Figure 5:**
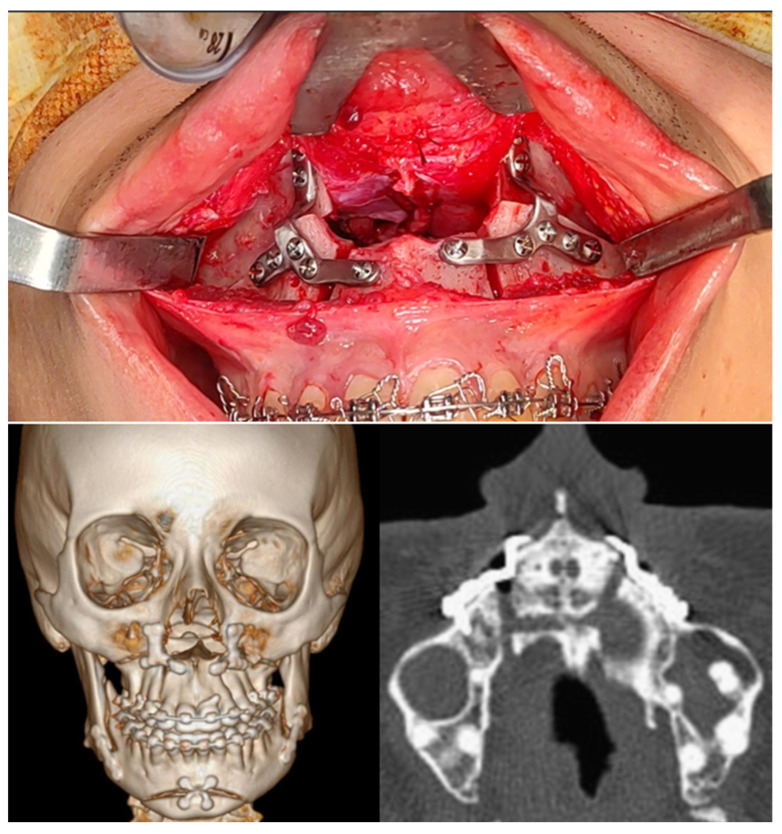
Intraoperative view of a three-piece Le Fort I osteotomy stabilized with an anterior support plate, and corresponding postoperative 3D reconstruction and axial images.

In selected cases, segmental Le Fort I osteotomies were performed to correct transverse discrepancies or anterior open bite, in line with previously described techniques [6]. The surgical sequence for segmental cases was standardized as follows: Guided Osteotomies: Initial Le Fort I and interdental segmental osteotomies were performed following the 3D-printed cutting guides, which also served to pre-drill the screw trajectories. Mobilization: Down-fracture was executed to ensure complete maxillary mobilization. Segmental Disjunction: Final separation of the maxillary segments was achieved using a fine osteotome to minimize bone loss at the interdental sites. Fixation of the Customized System: The patient-specific plates were first secured to the dentate and mobile segments (inferior) to move them in blocks. Repositioning: the inferior segments were then repositioned and fixated to the cranial segment using monocortical screws, ensuring the intraoperative transfer of the pre-planned three-dimensional position (Figure 4 and Figure 5).

Mandibular bilateral sagittal split osteotomies (BSSOS) and, when indicated, genioplasty were subsequently performed to achieve final occlusion and facial symmetry. Intermaxillary fixation was temporarily used during verification and released after osteosynthesis. The postoperative dietary regimen was identical for both study groups, following a standardized institutional protocol designed to protect the bone–screw interface during the early phases of osseous consolidation. Weeks 1–6: Patients were restricted to a strict pureed (full liquid/blenderized) diet to eliminate all masticatory loading on the maxillary segments. Week 7 to Month 3: Progression to a soft mechanical diet was permitted, allowing for the intake of soft foods that require minimal chewing, while still avoiding hard or crunchy substances. Month 4 onwards: Patients were transitioned to an unrestricted, normal diet as clinical and radiographic evidence typically suggests adequate bone union by this stage.

### 2.3. Radiological Assessment, Outcomes, and Study Variables

**Figure 6:**
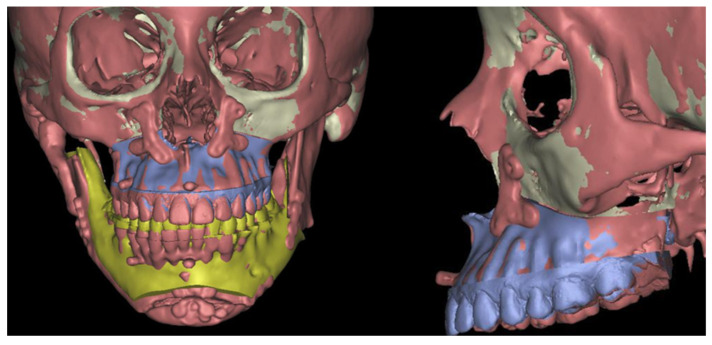
Superimposition analysis of preoperative virtual planning and post-operative CT scan.

High-resolution CT scans were acquired preoperatively and at 6 months postoperatively using standardized head positioning and occlusal registration. Image sets were converted to DICOM format and superimposed using voxel-based registration of the anterior cranial base to ensure consistent spatial alignment [7,8] (Figure 6).

The dependent variable was postoperative maxillary stability, defined as the three-dimensional linear displacement (mm) between pre- and postoperative positions at the following landmarks: right and left canines (MxC), right and left central incisors (MxI), right and left first molars (MxM), and the interincisal point.

Independent variables were as follows: (1) the number of maxillary segments (monoblock vs. ≥2 segments), and (2) type of customized plate (conventional vs. minimally invasive).

### 2.4. Statistical Analysis

All analyses were performed using IBM SPSS Statistics version 24.0 (IBM Corp., Armonk, NY, USA). Normality of the data distribution was evaluated using the Shapiro–Wilk test. Shapiro–Wilk testing confirmed non-normal distribution (*p* < 0.05) for all variables.

Since the data showed a non-normal distribution, descriptive statistics were expressed as median and interquartile range (IQR). Group comparisons for continuous variables were conducted using the Mann–Whitney U test, and categorical data were analyzed using Fisher’s exact test. All hypothesis tests were 2-sided, and a *p*-value < 0.05 was considered statistically significant.

## 3. Results

**Figure 7:**
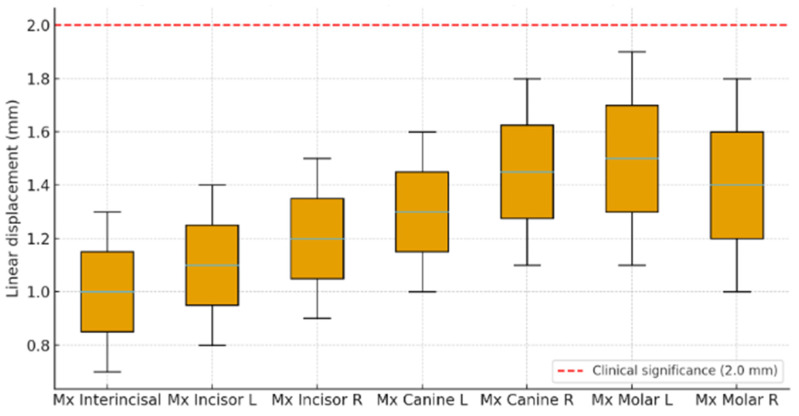
Distribution of 6-month maxillary landmark displacements (box-and-whisker plots) with the clinical threshold at 2.0 mm.

A total of 64 patients were included in the final analysis: 17 men and 47 women. At six months postoperatively, 18 out of 64 patients (28.1%) exhibited a displacement ≥ 2.0 mm in at least one maxillary reference point. The median linear displacement across all landmarks ranged between 0.7 and 4.28 mm. Variability was assessed via the interquartile range (IQR), showing consistent stability across landmarks (Figure 7). Maximum observed values exceeded 3 mm only in isolated cases, mostly at the canines and molars.

Clinically significant displacement (≥2 mm) was more frequent in the posterior and canine regions (Table 1). The left molar (20.6%), right molar (14.7%), right canine (13.2%), and left canine (11.8%) were the sites most commonly affected, while the interincisal and incisor points showed minimal movement.

When patients were grouped according to segmentation (1 segment vs. ≥2 segments), clinical instability (≥2 mm) was found in 10/41 (24.4%) and 10/27 (37.0%) patients, respectively. The absolute risk difference was 12.6 percentage points (95% CI: −12.3 to 37.5), and Fisher’s exact test did not reach statistical significance (*p* = 0.28). Regarding the magnitude of displacement, the Mann–Whitney U test showed comparable values between groups for most landmarks. The only exception was the left maxillary canine, which exhibited significantly higher displacement in the multisegmented group (Mann–Whitney U = 296; *p* = 0.027). All remaining points showed no significant differences (*p* > 0.05) (Table 2).

The type of customized plate (minimally invasive vs. conventional) showed no significant influence on postoperative stability. Clinically relevant displacement (≥2 mm) was detected in 28.6% of patients treated with minimally invasive plates and 33.3% with conventional plates (Fisher’s exact test *p* = 0.88). The calculated odds ratio for exceeding 2 mm was 1.23 (95% CI: 0.32–4.65), confirming the absence of association (Table 3).

## 4. Discussion

The present study evaluated the three-dimensional postoperative stability of the maxilla in patients who underwent orthognathic surgery using customized fixation plates designed through virtual surgical planning and CAD/CAM technology. At the six-month follow-up, the maxilla demonstrated overall skeletal stability, with displacements of less than 2 mm in most reference points analyzed. Only the right canine exhibited a greater degree of movement in multisegmental cases. No statistically significant differences were observed between conventional and minimally invasive customized plates, nor in the magnitude of displacement according to the number of maxillary segments. These findings suggest that patient-specific plates provide adequate postoperative skeletal stability of the maxilla, regardless of plate design. These results reinforce the paradigm that the reliability of PSI lies in the passive fit and pre-drilled trajectory rather than the sheer volume of titanium used [10].

Regarding the use of customized plates compared with conventional osteosynthesis, previous evidence has shown that both fixation techniques yield equivalent outcomes [10,18]. In other words, patient-specific osteosynthesis achieves comparable levels of three-dimensional stability, and in some cases, even smaller displacement ranges than conventional miniplate systems. Van der Wel et al. (2023) reported, in a randomized controlled trial comparing postoperative stability at one year between conventional and patient-specific osteosynthesis in splintless Le Fort I osteotomies, equivalent results for both techniques, with median translational changes of less than 1 mm and rotational deviations below 1° [10]. This finding supports the validity of the present results, in which the magnitude of postoperative displacements was also clinically negligible, remaining within the range considered stable in the literature (<2 mm). A critical analysis of our data reveals that while median values were stable, approximately 29% of patients exhibited point-specific displacements exceeding 2.0 mm, predominantly in the posterior maxilla. This rate is comparable to the long-term settling reported in waferless protocols by Van der Wel et al. (2023) [10].

The results regarding the type of customized plate (minimally invasive vs. conventional) showed that clinically relevant displacements occurred less frequently with minimally invasive plates than with conventional ones; however, this difference did not have a statistically significant effect on postoperative maxillary stability. The transition toward simplified fixation protocols is rooted in the biomechanical adequacy of the paranasal region. Mavili et al. (2009) demonstrated that two-plate fixation at the nasomaxillary buttress provides sufficient stability to prevent anteroposterior maxillary movement [19], a concept further validated by Susarla et al. (2020), who reported stable outcomes over a one-year follow-up following two-point nasomaxillary fixation in maxillary advancements [20]. Furthermore, comparative studies by Beyler et al. (2021) confirmed that bilateral fixation at the piriform aperture yields postoperative stability comparable to traditional four-plate systems, with no significant differences in vertical or sagittal relapse rates [21]. Building upon these principles, the integration of patient-specific implants (PSI) offers a superior alternative to conventional hardware. Alfaro et al. (2024) introduced a two-plate patient-specific osteosynthesis (PSO) system specifically designed to facilitate minimally invasive approaches, highlighting advantages such as reduced operative time and decreased surgical morbidity without compromising accuracy [22]. Our study reinforces this paradigm shift, demonstrating that minimally invasive customized plates, limited to the nasomaxillary buttress, do not increase the risk of skeletal instability compared to conventional extended designs. Consequently, the use of two anteriorly placed customized plates appears to be a robust and less invasive alternative for maintaining three-dimensional maxillary stability in Le Fort I osteotomies. Also, unlike conventional plates that require manual bending, which can introduce metal fatigue and internal stresses, these patient-specific implants are fabricated via selective laser melting to match the unique anatomical contours of the patient. This precise adaptation ensures that functional loads are distributed more uniformly across the bone–screw interface from the immediate postoperative period. As demonstrated by Stokbro et al. (2020), the absence of pre-surgical metal fatigue and the improved passive fit of printed plates provide superior rigidity and resistance to displacement compared to manually bent systems [23].

To our knowledge, this is one of the few studies comparing “minimally invasive” paranasal customized plates versus extended buttress plates. We found no statistically significant difference in stability between the two designs (OR 1.23; *p* = 0.878). Also, our findings support the biomechanical findings of Stokbro et al. (2020), which demonstrated in an in vitro model that patient-specific printed plates provide superior rigidity and resistance to displacement compared to manually bent conventional plates, mainly due to the absence of pre-surgical metal fatigue and improved passive fit [23].

Similarly, Hanafy et al. (2020), in their randomized controlled clinical trial comparing a fully digital computer-guided workflow (CAD/CAM) with a mixed analogue–digital approach, found that the computer-guided group showed significantly more accurate plan transfer for all linear and angular measurements when compared to the classic occlusal wafer group [12]. These findings confirm that a fully digital workflow provides greater accuracy in transferring the virtual surgical plan to the operative field [12]. Therefore, it can be inferred that the use of digitally designed cutting guides and patient-specific osteosynthesis systems enhances intraoperative precision, minimizes human error associated with manual plate bending, and contributes to a more predictable and reliable surgical outcome.

Segmental stability remains a critical biomechanical challenge in maxillary surgery. We observed a statistically significant increase in displacement at the canine level in multi-segment osteotomies (*p* = 0.041). This localized instability is likely attributable to the reduced bone contact area in the interdental osteotomy sites and the transverse relapse forces from palatal soft tissue stretching. While PSI plates provide rigid external fixation, they cannot fully counteract the elastic recoil of the palatal mucosa in large expansions without adequate consolidation time. This finding advocates for the potential need for auxiliary trans-palatal stabilization or extended consolidation periods in cases of major transverse segmentation, even when PSI is used.

Finally, it is important to consider the role of bone grafting in maxillary orthognathic surgery, which was considered on a case-by-case basis. While not routinely performed in our cohort, we acknowledge its importance in patients with significant maxillary down grafting or intersegmental gaps. In such scenarios, bone grafts or bone substitutes act as a biological scaffold to facilitate primary bone healing and minimize the risk of skeletal relapse. In multi-segment cases where localized instability was noted at the osteotomy sites, the use of grafting material could provide additional structural support.

## 5. Conclusions

In conclusion, customized maxillary plates offer reliable 3D stability. The reduction in the plate’s footprint does not increase the risk of relapse, supporting the use of minimally invasive designs. However, careful attention is required in multi-segment cases, where localized instability at the osteotomy sites remains a challenge independent of the fixation method.

## Figures and Tables

**Table 1:** Frequency of clinically significant displacement (≥2 mm) by landmark (n = 64).

Landmark	n (%) Exceeding 2 mm
Maxillary molar (L)	14 (20.6%)
Maxillary molar (R)	10 (14.7%)
Maxillary canine (R)	9 (13.2%)
Maxillary canine (L)	8 (11.8%)
Maxillary incisors (R + L)	5 < 10%
Inter-incisal point	2 < 10%

**Table 2:** Comparison of 6-month maxillary landmark displacement between single-segment and multisegment Le Fort I osteotomies.

Landmark	Single Segment Median (n = 43)	Multisegmented Median (n = 21)	Mann–Whitney U (W)	*p*-Value
Inter-incisal point	0.90 mm	1.09 mm	414	0.596
Incisor (L)	0.87 mm	1.08 mm	410	0.557
Incisor (R)	0.88 mm	1.08 mm	439	0869
Canine (L)	0.89 mm	1.43 mm	296	0.027
Canine (R)	0.89 mm	1.20 mm	338	0.107
Molar (L)	1.06 mm	1.37 mm	323	0.068
Molar (R)	0.96 mm	1.20 mm	372	0.261

**Table 3:** Association between customized plate design and clinical instability (≥2.0 mm at any landmark).

Plate Type	≥2 mm (n/%)	OR (95% CI)	Fisher *p*
Minimally invasive (n = 41)	12/29.3%	1.17 (0.37–3.70)	1.00
Conventional (n = 23)	6/26.1%	Reference	–

## Data Availability

Any inquiries regarding this study should be requested to the corresponding author.

## Abbreviations

The following abbreviations are used in this manuscript:

VSP	Virtual Surgical Planning
CAD/CAM	Computer-Aided Design and Manufacturing
DICOM	Digital Imaging and Communications in Medicine
